# Vibration Sensor Monitoring of Nickel-Titanium Alloy Turning for Machinability Evaluation

**DOI:** 10.3390/s17122885

**Published:** 2017-12-12

**Authors:** Tiziana Segreto, Alessandra Caggiano, Sara Karam, Roberto Teti

**Affiliations:** 1 Fraunhofer Joint Laboratory of Excellence on Advanced Production Technology (Fh-J_LEAPT Naples), P.le Tecchio 80, 80125 Naples, Italy; alessandra.caggiano@unina.it (A.C.); roberto.teti@unina.it (R.T.); 2 Department of Chemical, Materials and Industrial Production Engineering, University of Naples Federico II, P.le Tecchio 80, 80125 Naples, Italy; 3 Department of Industrial Engineering, University of Naples Federico II, P.le Tecchio 80, 80125 Naples, Italy; 4 South Eastern Applied Material Research Centre, WIT, Applied Technology Building, Paddy Browns Road, X91 TX03 Waterford, Ireland; karam.sarah@gmail.com

**Keywords:** Nickel-Titanium alloy, turning, sensor monitoring, vibration, machinability, cognitive pattern recognition

## Abstract

Nickel-Titanium (Ni-Ti) alloys are very difficult-to-machine materials causing notable manufacturing problems due to their unique mechanical properties, including superelasticity, high ductility, and severe strain-hardening. In this framework, the aim of this paper is to assess the machinability of Ni-Ti alloys with reference to turning processes in order to realize a reliable and robust in-process identification of machinability conditions. An on-line sensor monitoring procedure based on the acquisition of vibration signals was implemented during the experimental turning tests. The detected vibration sensorial data were processed through an advanced signal processing method in time-frequency domain based on wavelet packet transform (WPT). The extracted sensorial features were used to construct WPT pattern feature vectors to send as input to suitably configured neural networks (NNs) for cognitive pattern recognition in order to evaluate the correlation between input sensorial information and output machinability conditions.

## 1. Introduction

Nickel-Titanium (Ni-Ti) alloys are recognized for their excellent electrical, mechanical and damping properties, including superelasticity and shape-memory. Such attributes make these alloys a promising material for a number of applications in different fields like automotive, aerospace and robotics [1,2,3].

For instance, as Ni-Ti alloys possess high strength, strong corrosion resistance as well as excellent thermal fatigue properties and thermal stability, they have been extensively applied for engine parts production, such as aircraft engine compressor disks, turbine disks, bearing rings, turbine blades and other parts working at high temperature [4].

More recently, Ni-Ti intermetallic compounds, also known with the name of Nitinol, have been introduced in the biomedical sector for the fabrication of coronary stents and orthodontics and orthopedic implants due to the high biocompatibility of the metal alloy [5,6].

However, due to the high temperatures and stresses generated during machining of Ni-Ti alloys, the latter are classified as difficult-to-machine materials. A rapid tool failure and poor surface quality of the workpiece are generated during Ni-Ti alloys machining due to excessive burr formation, adhesions on the machined surface and microstructure alterations of the workpiece material [7]. Moreover, the microstructure of the bulk material subsurface is altered during machining due to plastic deformation and white layer formation [8].

Diverse research studies discussed the challenges related to machining of Ni-Ti alloys, focusing on process parameters optimization, tool wear identification or workpiece surface quality.

The study in [9] focused on machinability and surface integrity in milling of Nitinol alloy, with the objective to investigate the dynamic mechanical behavior of Nitinol in cutting, as well as to explore the tool wear mechanisms and examine the process-induced surface integrity and edge quality.

Tool wear behaviour and cutting forces in machining of Ni-Ti shape memory alloys under various machining conditions (dry, preheated, and cryogenic cooling) and different cutting speeds were investigated in [10]. 

The research study in [11] demonstrated how machining of Ni-Ti alloys under different cutting and cooling conditions affects their resulting surface integrity characteristics, including surface quality, topography and microstructure.

The machinability of Ni-Ti based shape memory alloys with reference to turning and drilling processes was examined in [12] by varying process parameters such as cutting speed, feed rate or cooling lubricant method, showing that the machinability distinctly depends on cutting speed and feed rate, for which higher values than those mostly recommended in the literature should be selected. A persisting problem in the case of turning is related to the poor chip breakage and burr formation caused by the remarkably high ductility of these materials.

Turning of Ni-Ti alloys was also studied in [13], where the influence of the cutting tool material on the machining process was evaluated. Based on the experimental tests, the metal removal rate was significantly increased and the tool life was extended when utilizing coated cemented carbide tools.

An approach to improve the machinability of Ni-Ti alloys through the application of chilled air was studied in [14], showing that lower cutting forces, reduced burr height and lower tool wear can be achieved. In [15], the effects of cryogenic cooling on tool wear rate and surface quality were investigated by comparing the new findings from cryogenic machining with the results obtained under minimum quantity lubrication and dry machining conditions. 

An overview of the machinability of aerospace engine materials with emphasis on titanium and nickel-based alloys was presented in [16]. The enhancement of the machinability of these alloys at high speed conditions can be achieved through a proper combination of the appropriate tool material, machining technique and cooling technology.

To improve and optimize machining processes, a number of studies in the last decades proposed the use of on-line sensor systems for monitoring of tool conditions [17,18,19], machine tool state [20,21], chip formation [22,23], vibration control [24], chatter detection, surface integrity, process conditions, etc. [25,26,27]. However, only few papers in the literature tackled the specific issue of sensor monitoring applied to machining of Ni-Ti alloys [28]. 

To exploit the sensor monitoring results as a support to decision making systems for machining processes, the development and implementation of advanced sensor monitoring procedures, based on innovative technologies and approaches, is essential.

The sequence of activities to be performed in sensor monitoring of machining process conditions can be summarised as follows [29]:

- detection of machining process variables through sensorial perception methods;
- sensor signals processing;
- relevant sensorial features extraction and selection;
- implementation of decision making paradigms for diagnosis on machining process conditions;
- activation of corrective actions.

Usually, the sensor signals obtained during process monitoring are subjected to signal conditioning (filtering, amplification, A/D conversion, segmentation) and processing with the aim to generate functional signal features [20,25,26,27,28,29]. The main procedures for signal features extraction which are typically used in sensor monitoring research can be classified as follows: time-domain methods (e.g., principal component analysis (PCA)) [21,22,23]), frequency domain methods (e.g., fast Fourier transform (FFT)) [20,30]) and time-frequency domain methods (e.g., wavelet transform (WT) [31,32,33]).

The signal features extracted through these methods are then supplied to and evaluated by cognitive decision-making support systems based on approaches such as neural networks, fuzzy logic, genetic algorithms, etc. [34,35] for the final diagnosis on the process.

In this framework, the aim of this paper is to develop an advanced sensor monitoring procedure for turning of Ni-Ti alloys in order to realize a reliable and robust classification of the process quality in terms of machinability conditions.

The sensor monitoring procedure implemented during experimental turning tests on Nitinol bars is based on the on-line acquisition of vibration sensor signals, that include valuable information on the machining process.

Specifically, vibrations occurring during metal cutting can be classified into dependent and independent of the cutting process. The most renowned type of vibration in machining is chatter, i.e., self-excited vibration, which is detrimental to surface finish and tool life. Vibrations dependent on metal cutting can exhibit specific characteristics as a function of the process. In particular, tool engagement conditions during machining play a significant role in the vibration produced [7,36].

Due to the high correlation between produced vibrations and machining process characteristics, vibration signals have been successfully employed in the literature for tool condition monitoring applications and in-process prediction of surface roughness during turning processes [37,38,39].

Accordingly, with the aim to perform process monitoring in turning of Ni-Ti alloys, a vibration sensor system has been selected in this research work to acquire valuable sensor signals that can be correlated to the process conditions.

The detected vibration signals are processed through an advanced signal processing method in the time-frequency domain based on wavelet packet transform (WPT) [29,31]. The sensorial features extracted in this way are used to construct WPT feature pattern vectors to feed neural network (NN) based cognitive pattern recognition paradigms [40] able to find correlations between input sensorial information and output process quality in terms of machinability conditions [29,41].

## 2. Experimental Setup and Vibration Sensor Signals Acquisition

The experimental tests of Ni-Ti alloy turning for machinability assessment were performed on Nitinol (55% Ni, 45% Ti) bars of 40 mm diameter and 200 mm length. The selected cutting tools were coated carbide inserts, Kendex TPGN160308, with PVD TiAlN coating (KC5010 grade), rake angle: γ = 0°, clearance angle: α = 11°, edge length L10 = 16.5 mm, thickness S = 3.18 mm, nose angle = 60°, nose radius = 0.8 mm and no chip breaker. Different process conditions were tested using the following parameters: cutting speed, v_c_ = 40, 55, 75, 100, 130 m/min; feed rate, f = 0.10, 0.15, 0.20 mm/rev; depth of cut, a_p_ = 0.5 mm; coolant: KSM 950 (Hebro-Chemie, Mönchengladbach, Germany). The depth of cut, a_p_, was always equal to 0.5 mm, while cutting speed, v_c_, and feed, f, were varied among the tests. The experimental turning test programme with the relevant cutting parameters is reported in Table 1. By combining the three cutting parameters (v_c_, f and a_p_), a total of 15 turning tests were performed.

**Figure 1:**
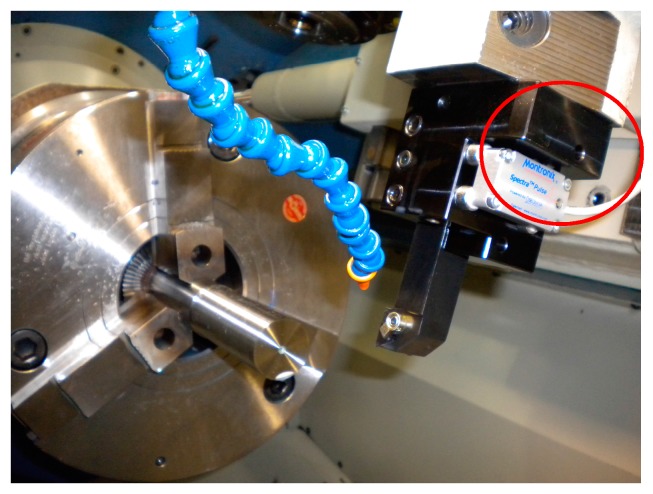
Three-axis vibration sensor mounted on the tool holder.

A sensor monitoring system endowed with a 3-axis wireless vibration sensor (Spectra Pulse wireless sensor by Montronix S.r.l. Vigevano (PV), Italy) was utilized to acquire the sensor signals relative to the 3 acceleration components a_x_, a_y_, and a_z_ along the x, y, and z directions (Figure 1). The 3-axis wireless vibration sensor is provided with a magnetic base and was mounted on the tool holder without need for bolting. This sensor is a miniature monitoring system, utilizing MEMS technology, which incorporates sensor, processor, amplifier, memory and A/D conversion, and directly feeds the digitised signals to the PC via wireless network communication. During the machining tests, the a_x_, a_y_, and a_z_ vibration acceleration components were acquired and digitised by the wireless vibration sensor at 3 kHz sampling rate.

## 3. Vibration Sensor Signal Processing

The acquired vibration signals were processed through advanced methods of signal processing for feature extraction and cognitive pattern recognition with the aim to identify the correlations between sensorial data and process conditions during turning of Nitinol [25,29]. 

**Figure 2:**
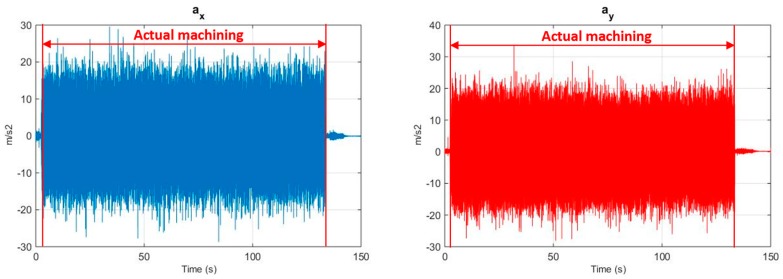

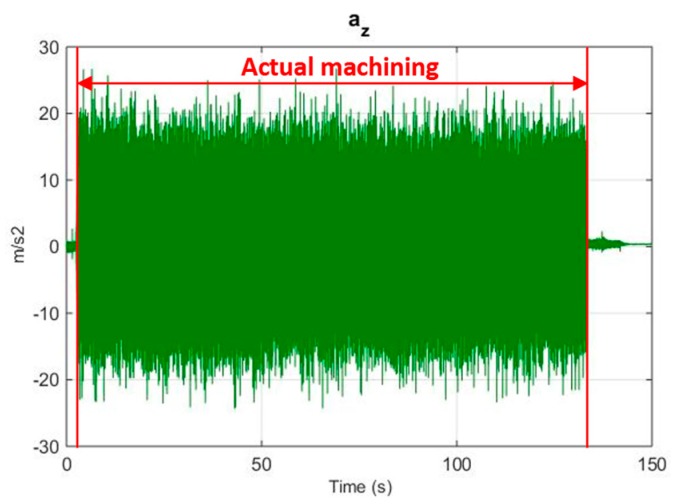
Vibration acceleration components signals acquired during turning with v_c_ = 40 m/min, f = 0.10 mm/rev, a_p_ = 0.5 mm. Vertical red lines delimit the signal portion relative to actual machining.

The three vibration acceleration components a_x_, a_y_, and a_z_ detected by the wireless vibration sensor were symmetric about their average value which was differently shifted for each vibration acceleration component with respect to 0 m/s^2^. This phenomenon is due to the influence exerted on the vibration acceleration components by the gravity acceleration along the x, y and z axes of the vibration sensor, which varies depending on the sensor orientation (their sum is 9.81 m/s^2^). Initially, before signal segmentation, a pre-processing procedure was applied to remove the offset of each acquired signal with respect to the zero axis. This offset was calculated for each vibration acceleration component as the signal average before the actual machining start; such average was then subtracted to the corresponding signal. Then, the signals were processed by cutting off the signal portions corresponding to the transient conditions (i.e., beginning and end of the turning test). Figure 2 shows the a_x_, a_y_, and a_z_ vibration acceleration signals for the turning test carried out with v_c_ = 40 m/min, f = 0.10 mm/rev, a_p_ = 0.5 mm, where the signal portion to be considered for further analysis (corresponding to actual machining) is delimited by vertical red lines. An automatic cut off procedure was carried out by calculating the square of each signal and using an experimentally determined threshold (equal to 20 m/s^2^) to identify the beginning and end of the signal relative to actual machining.

Afterwards, all the cut signals were reduced to a common minimum length (50,000 samples) to obtain the pre-processed vibration acceleration signals. The latter signals were subdivided into five equal parts of 10,000 samples for further signal analysis, obtaining a total of 75 vibration acceleration signal specimens for each a_x_, a_y_, and a_z_ component.

## 4. Feature Extraction through Wavelet Packet Transform

**Figure 3:**
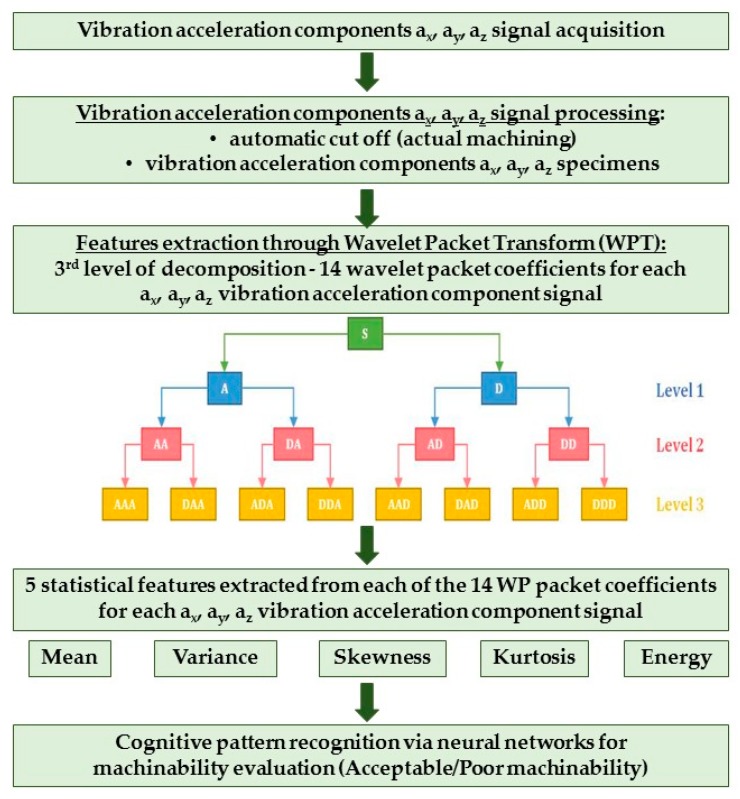
Features extraction through wavelet packet transform (WPT) and pattern feature vectors construction.

From the pre-processed vibration acceleration signal specimens, features capable of adequately describing the signal and maintaining the relevant information about the process were extracted utilizing the wavelet packet transform (WPT) method in the time-frequency domain (Figure 3).

Generally, the basis function of wavelet transform (WT) are small waves (wavelets) of varying frequency and limited duration; in this way, the signal is represented as a superposition of wavelets. Therefore, the WT can extract information in the time domain with reference to different frequency bands. This simultaneous time-frequency decomposition gives the WT a special advantage over the traditional Fourier transform in analysing non-stationary signals [31,32,33].

The WPT is a generalization of wavelet decomposition that provides level by level transformation of a signal from the time domain into the frequency domain. In the decomposition procedure, high-pass and low-pass filters are utilized to decompose an incoming signal S (Figure 3). At the first level, the output from the low-pass filter represents the “approximation” (A) of the signal and the output from the high-pass filter represent the “detail”, D. Proceeding to the second level, each approximation and detail packet is split again into further approximations, AA and AD, and details, DA and DD. After decomposition, the original signal S can be represented as the summation of the wavelet packets. To perform WPT, a mother or basis wavelet is first selected among different wavelet filter families. The signal is then decomposed to a set of scaled and translated versions of the mother wavelet. The translation and scaling operations applied to the mother wavelet are performed to calculate the wavelet packet coefficients, which represent the correlation between the wavelet and a localised section of the signal. The wavelet packet coefficients are calculated for each wavelet segment [42]; they are used to scale and shift the mother wavelet and are capable of relating the mother wavelet with the original signal. These coefficients can be processed to obtain statistical features to be used for pattern recognition procedures [31,32,33].

In this paper, the WPT of the a_x_, a_y_, and a_z_ vibration acceleration components was realized using the Daubechies db3 mother wavelet. The decomposition procedure, performed separately for a_x_, a_y_, and a_z_, was carried out up to the third level, generating a total of 14 wavelet packets of coefficients for each vibration acceleration component: for each packet, five statistical features, i.e., mean, variance, skewness, kurtosis, and energy, were calculated (Figure 3). These features, combined into pattern feature vectors, represent a useful input for the neural network based decision making algorithm aimed at finding correlations between input pattern feature vectors and output machinability classification.

**Figure 4:**
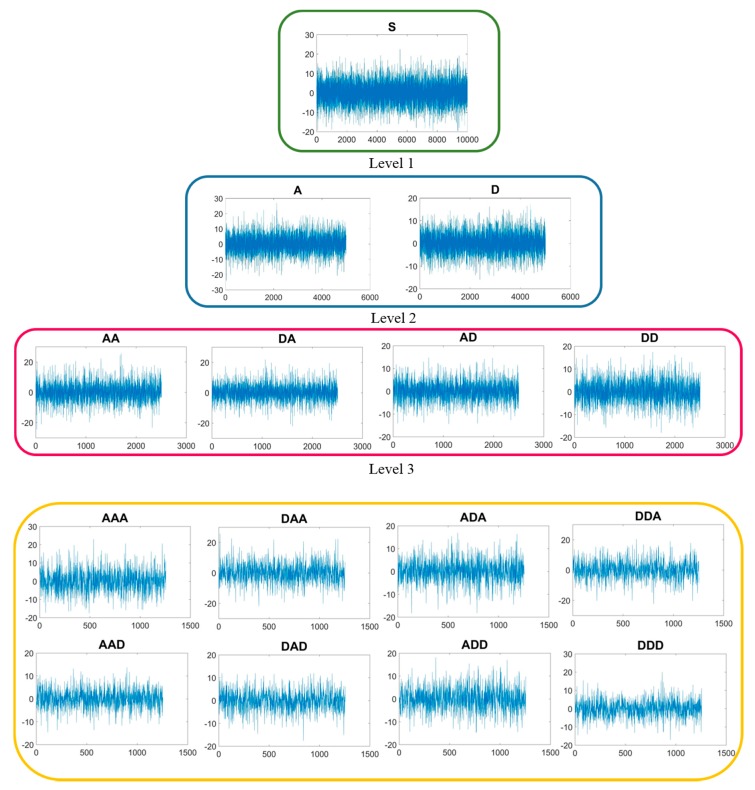
Three-level WPT decomposition for the first part (10,000 digital signal samples) of the a_x_ vibration acceleration signal for the Test 1.

The WPT signal decomposition procedure for the first part (10,000 digital signal samples) of the a_x_ vibration acceleration signal acquired during the Test 1 with v_c_ = 40 m/min, f = 0.10 mm/rev, a_p_ = 0.5 mm is shown in Figure 4. In the figure, all the 14 WPT packets of coefficients extracted from the signal are reported in separate charts showing how the original signal is modified by proceeding with the WPT processing. The signal feature extraction procedure performed on each packet of coefficients provides five statistical features (mean, variance, skewness, kurtosis, and energy) capable of adequately describing the signal and maintaining the relevant information about the process.

**Figure 5:**
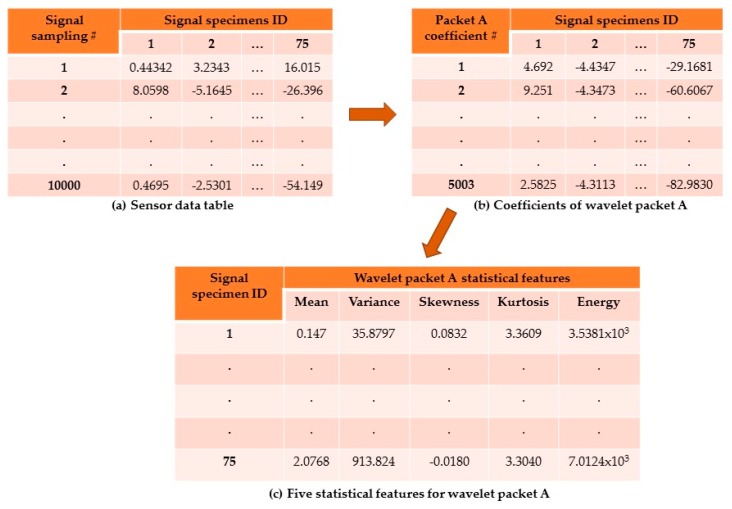
WPT feature extraction procedure for wavelet packet A of a_x_ vibration acceleration component: (**a**) sensorial data table; (**b**) calculation of packet A coefficients; (**c**) five statistical features for packet A.

As an example, the WPT feature extraction procedure for packet A of a_x_ is illustrated in Figure 5. To perform the WPT feature extraction procedure, a sensorial data table containing the n signals (75 columns) each composed of j digital signal samples (10,000 rows) was created as shown in Figure 5a. By applying the WPT to the 75 vibration acceleration signals, the corresponding 75 A packets (columns) consisting of 5003 coefficients (rows) were obtained (Figure 5b). For each A packet, the five statistical features (mean, variance, skewness, kurtosis, energy) were calculated starting from its 5003 coefficients (Figure 5c). Overall, 14 WPT pattern feature vectors (corresponding to the 14 WPT packets) for each of the three vibration acceleration components a_x_, a_y_, and a_z_, were obtained.

## 5. Machinability Classification

With the scope to assess the machinability corresponding to each turning test, three process quality parameters were taken into account: crater wear, flank wear, and machine vibrations level during machining. These three process quality parameters were ranked within a grading scale between 1 (good) and 5 (bad) on the basis of the expert knowledge of a skilled turning operator. In particular, the machine vibrations level was evaluated by an expert operator who carried out his assessment on the basis of his experience as well as with the support of a portable vibration measurement instrumentation. The flank wear and crater wear were categorized by the operator into the five different wear levels by taking into account as critical flank wear value VBmax = 0.6 mm indicated by the standard on tool life testing with single-point turning tool (ISO 3685:1993), and as critical crater wear value the crater front distance KF = 0.02 mm. 

The machinability was then classified as Acceptable or Poor according to the following rule: in case a ranking ≥ 4 was verified for any of the three quality parameters, then the machinability was classified as Poor. In all the other cases, it was considered Acceptable (Table 2). The machinability classification was utilized as output in the cognitive neural network pattern recognition procedure.

## 6. Cognitive Pattern Recognition via Neural Networks

The obtained WPT pattern feature vectors were utilised as input to diverse neural networks (NN) for cognitive pattern recognition (Figure 3) aimed at finding correlations between input pattern feature vectors and output machinability classification [34,41]. 

The NN implementation was carried out in the MatLab^®^ environment [43] using three-layer feed-forward back-propagation supervised network architectures for each of the 42 input pattern feature vectors (14 pattern feature vectors for each of the three vibration components) with the following structure:

- input layer with five nodes, according to the number of input feature vector elements (the five statistical features for each packet);
- hidden layer with a number of nodes equal to 5, 10 or 15;
- output layer with only one node, providing a binary target value associated to machinability condition: 0 = Acceptable; 1 = Poor.

The NN training set was built up by mating the correct binary target value to each of the 75 vibration acceleration signal specimens in order to map the input pattern feature vector to the output machinability condition: the 75 obtained training cases included 45 Acceptable and 30 Poor machinability cases. The NN training function was based on Levenberg–Marquardt algorithm with the following training parameters: the maximum number of the epochs was set at 1000, the performance goal was fixed to 0, the minimum performance gradient was equal to 1 × 10^−7^, and the maximum mu parameter value was 1 × 10^10^. The NN training stops as soon as one of these conditions occurs: the maximum number of epochs is reached, the performance is minimized to the goal, the performance gradient falls below the selected minimum, and mu exceeds its maximum.

The leave-k-out method (k = 1) was employed: one by one, each pattern feature vector was removed in turn from the training set for testing and the remaining patterns were used for training [22]. The success of the NN machinability classification was expressed on the basis of the value of the indicator E = (O_a_ − O_d_), where O_a_ is the actual output and O_d_ the desired output. The NN classification is considered correct if −0.5 ≤ E ≤ +0.5. The ratio of correct classifications over the total training cases yields the NN success rate (SR).

## 7. Results and Discussion

The NN performance expressed as SR (%) are shown in Table 3, Table 4 and Table 5 for each vibration acceleration component a_x_, a_y_, and a_z_, with reference to the diverse NN configurations (5-5-1, 5-10-1, 5-15-1) and all the 14 WPT pattern feature vectors. The NN SR was reported for the three diverse types of machinability classification: SR for the identification of Acceptable cases, SR for the identification of Poor cases, and overall SR corresponding to all cases (Acceptable + Poor).

Table 3 shows that, the NN SR for the a_x_ component ranges between 77.8–93.3% in the case of Acceptable machinability identification, 50.0–83.3% in the case of Poor machinability identification, and 68.0–88.0% for Overall cases. The best NN SR value (93.3%) obtained in the identification of Acceptable machinability conditions was obtained with the 5-5-1 NN configuration and the ADD WPT packet, while the best NN SR value (83.3%) is achieved for Poor machinability conditions with the same NN configuration (5-5-1) and WPT packet (DAD). The best NN SR value (88.0%) for Overall (all cases) machinability classification is obtained for the 5-5-1 NN configuration and the DAD WPT packet showing a good balance between the identification of Acceptable (SR = 91.1%) and Poor (SR = 83.3%) machinability conditions.

As regards the a_y_ vibration acceleration component (Table 4), the NN SR ranges between 75.6–97.8% in the case of Acceptable machinability, 50.0–80.0% in the case of Poor machinability identification, and 68.8–85.3% in the case of Overall machinability. The best NN SR value (85.3%) in terms of Overall classification is obtained for two NN configurations: 5-5-1 and 5-10-1. For both NN configurations, the WPT packet that performs best is the DDA. For the 5-5-1 NN configuration, a SR value equal to 97.8% is obtained in the identification of Acceptable machinability conditions, while a SR value equal to 66.7% is achieved in Poor machinability conditions. For the 5-10-1 NN configuration, the identification of Acceptable machinability conditions gives a SR value equal to 95.6%, while for Poor machinability conditions a SR value equal to 70.0% is achieved, showing a better performance than the 5-5-1 NN in the identification of Poor machinability conditions.

Table 5 shows that, the NN SR for the a_z_ component ranges between 77.8–97.8% for Acceptable machinability identification, 56.7–80.0% for Poor machinability identification, and 73.3–88.0% for Overall machinability identification. The best NN SR value (88.0%) in terms of Overall classification is obtained for two NN configurations: 5-10-1 and 5-15-1. For the 5-10-1 NN, the WPT packet that performs best is the ADA, giving a SR = 93.3% for Acceptable machinability and a SR = 80.0% for Poor machinability. For the 5-15-1 NN, the best performing WPT packets are the DA, giving a SR = 95.6% for Acceptable machinability and a SR = 76.7% for Poor machinability, and the DD with SR = 93.3% for Acceptable machinability and SR = 80.0% for Poor machinability. Thus, the 5-15-1 NN displays a better performance in the identification of Poor machinability conditions using the DD than when using the DA packet.

Summarizing, Table 3, Table 4 and Table 5 show that the NN SR values are always notably higher for the identification of Acceptable machinability conditions than for the Poor machinability conditions.

**Figure 6:**
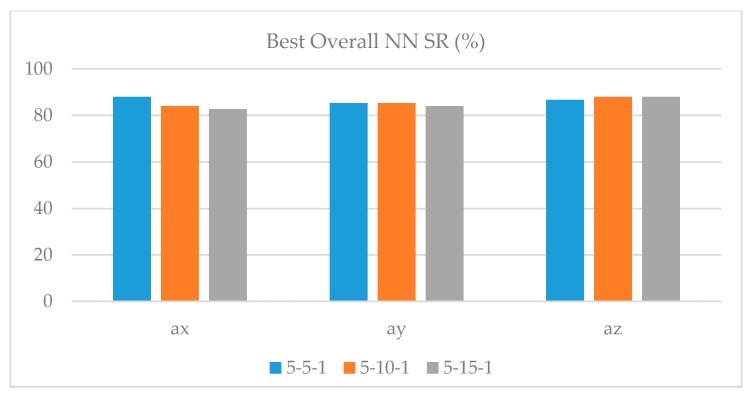
Best Overall NN SR for the a_x_, a_y_, and a_z_ vibration acceleration components using the three NN configurations.

In Figure 6, the best NN SR is reported for each vibration acceleration component and for the three diverse NN configurations. By comparing the performance of the three vibration acceleration components, a_x_, and a_z_ display the same behaviour with a maximum Overall SR equal to 88.0%. However, a_x_ exhibits a better balance in the identification of Acceptable and Poor machinability.

## 8. Conclusions

An on-line sensor monitoring procedure based on the acquisition of vibration sensor signals during turning of Ni-Ti alloys was implemented to achieve the in-process assessment of machinability conditions. The three vibration acceleration components a_x_, a_y_, and a_z_ were detected through a sensor monitoring system endowed with a three-axis wireless vibration sensor mounted on the tool holder in proximity of the tool insert.

An advanced signal processing method in the time-frequency domain, wavelet packet transform (WPT), was applied to the pre-processed vibration acceleration signals with the aim to extract relevant features able to maintain the relevant information about the process. These extracted features were used to construct WPT pattern feature vectors to feed to suitably configured supervised neural networks (NN) for the identification of machinability conditions.

The NN performance was classified in terms of the NN success rate (SR), i.e., the ratio of correct classifications over the total training cases. 

As regards the NN SR for each vibration acceleration component, it was shown that a_x_ and a_z_ give the same maximum overall SR = 88.0%, against the a_y_ maximum overall SR = 85.3%. Moreover, a_x_ showed the best balance in the identification of Acceptable (SR = 93.3%) and Poor (SR = 83.3%) machinability conditions.

As regards the performance of the 14 WPT pattern feature vectors used as input to the NN, the best results were obtained with the DAD pattern feature vectors for a_x_, the DDA pattern feature vectors for a_y_, and the ADA and DD pattern feature vectors for a_z_.

The obtained results showed that the NN SR for Acceptable machinability identification were always higher than for Poor machinability. As shown in Table 2, the number of NN training cases for Poor machinability is significantly lower than for Acceptable machinability (30 Poor machinability cases versus 45 Acceptable machinability cases). The lower success rate obtained in the case of the Poor machinability is related to the mentioned unbalanced training test cases. For this reason, as future developments of this paper, a new turning tests campaign will be considered in order to increase the number of training cases to be inputted to the NN. Moreover, future developments could involve the application of sensor fusion technology whereby the sensorial features are jointly extracted from the three a_x_, a_y_, and a_z_ vibration acceleration components and included together in sensor fusion pattern feature vectors to feed to the NN with enhanced synergical input information.

## Figures and Tables

**Table 1:** Experimental turning test programme.

Cutting Parameters	Experimental Test Values				
Cutting speed v_c_ (m/min)	40	55	75	100	130
Feed rate f (mm/rev)	0.10	0.15	0.20		
Depth of cut a_p_ (mm)	0.50				

**Table 2:** Machinability classification. Cutting speed is expressed in m/min and feed rate in mm/rev. Tool wear and vibrations level are ranked between 1 (good) and 5 (bad).

Test ID	Cutting Speed	Feed Rate	Flank Wear	Crater Wear	Vibrations Level	Overall Classification
**1**	40	0.10	1	1	2	Acceptable
**2**	40	0.15	1	1	2	Acceptable
**3**	40	0.20	1	1	3	Acceptable
**4**	55	0.10	1	1	2	Acceptable
**5**	55	0.15	1	1	3	Acceptable
**6**	55	0.20	1	1	3	Acceptable
**7**	75	0.10	2	2	2	Acceptable
**8**	75	0.15	1	1	3	Acceptable
**9**	75	0.20	1	1	4	Poor
**10**	100	0.10	1	1	2	Acceptable
**11**	100	0.15	1	1	4	Poor
**12**	100	0.20	1	1	4	Poor
**13**	130	0.10	5	5	3	Poor
**14**	130	0.15	5	4	4	Poor
**15**	130	0.20	5	2	5	Poor

**Table 3:** Neural network (NN) success rate (SR) (Acceptable, Poor, Overall = Acceptable + Poor) for vibration acceleration component a_x_ using NN configurations 5-5-1, 5-10-1, 5-15-1 and each of the 14 WPT packets.

Vibration Component—a_x_									
NN Configuration	5-5-1			5-10-1			5-15-1		
Wavelet Packet	Success Rates (%) for Features Extracted from Each WPT Packet								
	Acceptable	Poor	Overall	Acceptable	Poor	Overall	Acceptable	Poor	Overall
**A**	82.2	70.0	77.3	82.2	70.0	77.3	82.2	73.3	78.7
**D**	77.8	63.3	72.0	73.3	80.0	76.0	86.7	76.7	82.7
**AA**	84.4	73.3	80.0	80.0	63.3	73.3	84.4	60.0	74.7
**DA**	84.4	73.3	80.0	91.1	73.3	84.0	80.0	66.7	74.7
**AD**	77.8	70.0	74.7	82.2	66.7	76.0	82.2	63.3	74.7
**DD**	77.8	60.0	70.7	84.4	73.3	80.0	82.2	80.0	81.3
**AAA**	86.7	80.0	84.0	84.4	70.0	78.7	86.7	66.7	78.7
**DAA**	80.0	80.0	80.0	80.0	70.0	76.0	82.2	73.3	78.7
**ADA**	82.2	63.3	74.7	82.2	70.0	77.3	88.9	70.0	81.3
**DDA**	82.2	70.0	77.3	80.0	50.0	68.0	88.9	73.3	82.7
**AAD**	88.9	70.0	81.3	84.4	70.0	78.7	82.2	63.3	74.7
**DAD**	91.1	83.3	88.0	80.0	73.3	77.3	80.0	70.0	76.0
**ADD**	93.3	63.3	81.3	84.4	66.7	77.3	80.0	70.0	76.0
**DDD**	86.7	70.0	80.0	82.2	73.3	78.7	82.2	60.0	73.3

**Table 4:** NN SR (Acceptable, Poor, Overall = Acceptable + Poor) for vibration acceleration component a_y_ using NN configurations 5-5-1, 5-10-1, 5-15-1 and each of 14 the WPT packets.

Vibration Component—a_y_									
NN Configuration	5-5-1			5-10-1			5-15-1		
Wavelet Packet	Success Rates (%) for Features Extracted from Each WPT Packet								
	Acceptable	Poor	Overall	Acceptable	Poor	Overall	Acceptable	Poor	Overall
**A**	84.4	73.3	80.0	77.8	53.3	68.8	84.4	80.0	82.7
**D**	86.7	50.0	72.0	93.3	50.0	76.0	88.9	60.0	77.3
**AA**	86.7	66.7	78.7	82.2	63.3	74.7	86.7	60.0	76.0
**DA**	86.7	60.0	76.0	86.7	63.3	77.3	86.7	56.7	74.7
**AD**	88.9	56.7	76.0	93.3	56.7	78.7	91.1	60.0	78.7
**DD**	86.7	60.0	76.0	84.4	70.0	78.7	91.1	63.3	80.0
**AAA**	84.4	60.0	74.7	80.0	66.7	74.7	80.0	60.0	72.0
**DAA**	77.8	70.0	74.7	97.8	63.3	84.0	88.9	63.3	78.7
**ADA**	86.7	63.3	77.3	88.9	63.3	78.7	84.4	70.0	78.7
**DDA**	97.8	66.7	85.3	95.6	70.0	85.3	88.9	60.0	77.3
**AAD**	91.1	53.3	76.0	91.1	60.0	78.7	91.1	66.7	81.3
**DAD**	86.7	56.7	74.7	82.2	56.7	72.0	88.9	60.0	77.3
**ADD**	88.9	60.0	77.3	91.1	56.7	77.3	75.6	70.0	77.3
**DDD**	88.9	66.7	80.0	88.9	63.3	78.7	93.3	70.0	84.0

**Table 5:** NN SR (Acceptable, Poor, Overall = Acceptable + Poor) for vibration acceleration component a_z_ using NN configurations 5-5-1, 5-10-1, 5-15-1 and each of the 14 WPT packets.

Vibration Component—a_z_									
NN Configuration	5-5-1			5-10-1			5-15-1		
Wavelet Packet	Success Rates (%) for Features Extracted from Each WPT Packet								
	Acceptable	Poor	Overall	Acceptable	Poor	Overall	Acceptable	Poor	Overall
**A**	88.9	73.3	82.7	93.3	66.7	82.7	88.9	63.3	78.7
**D**	86.7	66.7	78.7	86.7	63.3	77.3	88.9	63.3	78.7
**AA**	86.7	76.7	82.7	88.9	63.3	81.3	91.1	66.7	81.3
**DA**	95.6	73.3	86.7	91.1	70.0	84.0	95.6	76.7	88.0
**AD**	86.7	60.0	76.0	86.7	73.3	78.7	86.7	63.3	77.3
**DD**	88.9	70.0	81.3	93.3	66.7	85.3	93.3	80.0	88.0
**AAA**	84.4	76.7	81.3	86.7	73.3	78.7	77.8	66.7	73.3
**DAA**	86.7	60.0	76.0	84.4	66.7	76.0	93.3	63.3	81.3
**ADA**	95.6	66.7	84.0	93.3	63.3	88.0	88.9	66.7	80.0
**DDA**	82.2	70.0	77.3	88.9	80.0	76.0	84.4	66.7	77.3
**AAD**	88.9	60.0	77.3	84.4	56.7	73.3	86.7	56.7	74.7
**DAD**	91.1	63.3	80.0	93.3	66.7	82.7	84.4	63.3	76.0
**ADD**	88.9	76.7	84.0	97.8	70.0	86.7	88.9	76.7	84.0
**DDD**	88.9	56.7	76.0	88.9	60.0	77.3	80.0	70.0	76.0

## References

[1] Shimizu K., Tadaki T., Funakubo H. (1987). Shape Memory Alloys.

[2] Duerig T.W., Melton K.N., Stöckel D., Wayman C.M. (1990). Engineering Aspects of Shape Memory Alloys.

[3] Turner J.D. (1994). Memory-metal Actuators for Automotive Applications. Proc. Inst. Mech. Eng..

[4] Zhu D., Zhang X., Ding H. (2013). Tool wear characteristics in machining of nickel-based superalloys. Int. J. Mach. Tools Manuf..

[5] Mussot-Hoinarda G., Elmay W., Peltier L., Laheurte P. (2017). Fatigue performance evaluation of a Nickel-free titanium-based alloy for biomedical application—Effect of thermomechanical treatments. J. Mech. Behav. Biomed. Mater..

[6] Fu C.H., Sealy M.P., Guo Y.B., Wei X.T. (2014). Austenite—Martensite phase transformation of biomedical Nitinol by ball burnishing. J. Mater. Process. Technol..

[7] Markopoulos A.P., Pressas I.S., Manolakos D.E. (2015). A Review on the machining of Nickel-Titanium shape memory alloys. Rev. Adv. Mater. Sci..

[8] Ulutan D., Özel T. (2011). Machining induced surface integrity in titanium and nickel alloys: A review. Int. J. Mach. Tools Manuf..

[9] Guo Y., Klink A., Fu C., Snyder J. (2013). Machinability and surface integrity of Nitinol shape memory alloy. CIRP Ann..

[10] Kaynak Y., Karaca H.E., Noebe R.D., Jawahir I.S. (2013). Analysis of Tool-wear and Cutting Force Components in Dry, Preheated, and Cryogenic Machining of NiTi Shape Memory Alloys. Procedia CIRP.

[11] Kaynak Y., Karaca H.E., Jawahir I.S. (2014). Surface integrity characteristics of NiTi shape memory alloys resulting from dry and cryogenic machining. Procedia CIRP.

[12] Weinert K., Petzoldt V. (2004). Machining of NiTi based shape memory alloys. Mater. Sci. Eng. A.

[13] Weinert K., Petzoldt V., Kötter D. (2004). Turning and Drilling of NiTi Shape Memory Alloys. CIRP Ann..

[14] Zailani Z.A., Mativenga P.T. (2016). Effects of Chilled Air on Machinability of NiTi Shape Memory Alloy. Procedia CIRP.

[15] Kaynak Y., Karaca H.E., Noebe R.D., Jawahir I.S. (2013). Tool-wear analysis in cryogenic machining of NiTi shape memory alloys: A comparison of tool-wear performance with dry and MQL machining. Wear.

[16] Ezugwu E.O., Bonney J., Yamane Y. (2003). An overview of the machinability of aeroengine alloys. J. Mater. Process. Technol..

[17] Caggiano A., Napolitano F., Teti R. (2017). Dry Turning of Ti6Al4V: Tool Wear Curve Reconstruction Based on Cognitive Sensor Monitoring. Procedia CIRP.

[18] Dimla D.E. (2000). Sensor signals for tool-wear monitoring in metal cutting: A review of methods. Int. J. Mach. Tools Manuf..

[19] Papacharalampopoulos A., Stavropoulos P., Doukas C., Foteinopoulos P., Chryssolouris G. (2013). Acoustic emission signal through turning tools: A computational study. Procedia CIRP.

[20] Er P.V., Teo C.S., Tan K.K. (2016). Approach towards sensor placement, selection and fusion for real-time condition monitoring of precision machines. Mech. Syst. Signal Process..

[21] Aouabdi S., Taibi M., Bouras S., Boutasseta N. (2017). Using multi-scale entropy and principal component analysis to monitor gears degradation via the motor current signature analysis. Mech. Syst. Signal Process..

[22] Segreto T., Simeone A., Teti R. (2014). Principal component analysis for feature extraction and NN pattern recognition in sensor monitoring of chip form during turning. CIRP J. Manuf. Sci. Technol..

[23] Segreto T., Simeone A., Teti R. (2012). Chip form classification in carbon steel turning through cutting force measurement and principal components analysis. Procedia CIRP.

[24] Yuan Y., Zhang H.T., Wu Y., Zhu T., Ding H. (2017). Bayesian learning-based model predictive vibration control for thin-walled workpiece machining processes. IEEE/ASME Trans. Mechatron..

[25] Teti R., Jemielniak K., O’Donnell G., Dornfeld D. (2010). Advanced monitoring of machining operations. CIRP Ann..

[26] Lauro C.H., Brandão L.C., Baldo D., Reis R.A., Davim J.P. (2014). Monitoring and processing signal applied in machining processes—A review. Measurement.

[27] Möhring H.-C., Litwinski K.M., Gümmer O. (2010). Process monitoring with sensory machine tool components. CIRP Ann. Manuf. Technol..

[28] Segreto T., Caggiano A., Teti R. (2015). Neuro-fuzzy system implementation in multiple sensor monitoring for Ni-Ti alloy machinability evaluation. Procedia CIRP.

[29] Teti R. (2015). Advanced IT methods of signal processing and decision making for zero defect manufacturing in machining. Procedia CIRP.

[30] Wang H., To S., Chan C.Y. (2013). Investigation on the influence of tool-tip vibration on surface roughness and its representative measurement in ultra-precision diamond turning. Int. J. Mach. Tools Manuf..

[31] Segreto T., Karam S., Simeone A., Teti R. (2013). Residual stress assessment in Inconel 718 machining through wavelet sensor signal analysis and sensor fusion pattern recognition. Procedia CIRP.

[32] Viktor Skrickij V., Bogdevičius M., Junevičius R. (2016). Diagnostic features for the condition monitoring of hypoid gear utilizing the wavelet transform. Appl. Acoust..

[33] Xu S., Jiang X., Huang J., Yang S., Wang X. (2016). Bayesian wavelet PCA methodology for turbomachinery damage diagnosis under uncertainty. Mech. Syst. Signal Process..

[34] Segreto T., Teti R. (2007). Applications of intelligent sensor monitoring for machining processes. Laser Metrology & Machine Performance VIII; Proceedings of the 8th International Conference on Laser Metrology, Machine Tool, CMM & Robotic Performance; Lamdamap 2007, Cardiff, UK, 28 June 2007.

[35] Wang L., Gao R.X. (2006). Condition Monitoring and Control for Intelligent Manufacturing.

[36] Peng Z., Chu F., He Y. (2002). Vibration signal analysis and feature extraction based on reassigned wavelet scalogram. J. Sound Vib..

[37] Upadhyay V., Jain P.K., Mehta N.K. (2013). In-process prediction of surface roughness in turning of Ti–6Al–4V alloy using cutting parameters and vibration signals. Measurement.

[38] Abouelatta O.B., Madl J. (2001). Surface roughness prediction based on cutting parameters and tool vibrations in turning operations. J. Mater. Process. Technol..

[39] Dimla D.E. (2002). The correlation of vibration signal features to cutting tool wear in a metal turning operation. Int. J. Adv. Manuf. Technol..

[40] Bishop C.M. (1995). Neural Networks for Pattern Recognition.

[41] D’Addona D., Segreto T., Simeone A., Teti R. (2011). ANN tool wear modelling in the machining of Nickel superalloy industrial products. CIRP J. Manuf. Sci. Technol..

[42] Gokhale M.Y., Khanduja D.K. (2010). Time domain signal analysis using wavelet packet decomposition approach. Int. J. Commun. Netw. Syst. Sci..

[43] Misiti M., Misiti Y., Oppenheim G., Poggi J.M. (2014). (1997–2014) Wavelet Toolbox for Use with MATLAB.

